# Racial Capitalism: A Fundamental Cause of Novel Coronavirus (COVID-19) Pandemic Inequities in the United States

**DOI:** 10.1177/1090198120922942

**Published:** 2020-04-26

**Authors:** Whitney N. Laster Pirtle

**Affiliations:** 1 University of California Merced, Merced, CA, USA

**Keywords:** capitalism, coronavirus, COVID-19, fundamental causes, health inequities, racism

## Abstract

Racial capitalism is a fundamental cause of the racial and socioeconomic inequities within the novel coronavirus pandemic (COVID-19) in the United States. The overrepresentation of Black death reported in Detroit, Michigan is a case study for this argument. Racism and capitalism mutually construct harmful social conditions that fundamentally shape COVID-19 disease inequities because they (a) shape multiple diseases that interact with COVID-19 to influence poor health outcomes; (b) affect disease outcomes through increasing multiple risk factors for poor, people of color, including racial residential segregation, homelessness, and medical bias; (c) shape access to flexible resources, such as medical knowledge and freedom, which can be used to minimize both risks and the consequences of disease; and (d) replicate historical patterns of inequities within pandemics, despite newer intervening mechanisms thought to ameliorate health consequences. Interventions should address social inequality to achieve health equity across pandemics.

Racial capitalism is a *fundamental cause* of disease in the world and will be a root cause of the racial and socioeconomic inequities in COVID-19 that we will be left to sort out when the dust settles. What is a fundamental cause? In Link and Phelan’s widely cited (1995) theoretical article, they argued that a social condition is a basic, fundamental cause of disease disparities if it (a) influences multiple disease outcomes, (b) affects disease outcomes through multiple risk factors, (c) involves access to flexible resources that can be used to minimize both risks and the consequences of disease, and (d) is reproduced overtime through the continual replacement of intervening mechanisms.

Sociological health research has since proven that both socioeconomic and racial social inequities are social conditions that fit the formula and contribute to socioeconomic and racial health inequities (i.e., Gee & Ford, 2011; Lutfey & Freese, 2005; Phelan & Link, 2015; Phelan et al., 2010; Sewell, 2016; Williams & Collins, 2001). I extend this conversation by arguing that the research is actually capturing how *racial capitalism* works to have a fundamental impact on health inequities, as Black radical thought traditions suggested as much decades ago. As introduced by Robinson (1983), racial capitalism is the idea that racialized exploitation and capital accumulation are mutually constitutive. Racial capitalism created the modern world system, through slavery, colonialism, and genocide because “the development, organization, and expansion of capitalist society pursued essentially racial directions, so too did social ideology” (Robinson, 1983, p. 2). Racially minoritized and economically deprived groups face capitalist and racist systems that continue to devalue and harm their lives, even within newer, supposedly deracialized neoliberal agendas (Clarno, 2017; Johnson, 2017).

We have ample evidence of racial capitalism as a cause of health inequities in the United States though, collectively, scholarship has not always connected all the pieces. For instance, Pulido (2016) argues that racial capitalism is at the very core of the Flint, Michigan lead water crisis:

The people of Flint are so devalued that their lives are subordinated to the goals of municipal fiscal solvency . . . this devaluation is based on both their blackness and their surplus status, with the two being mutually constituted. (p. 1)

Additional research has found that exposure to the Flint water crisis has been linked to both physical (Sadler et al., 2017) and mental (Cuthbertson et al., 2016) health problems for poor and people of color and can be explained through multiple mechanisms, such as disinvested, racially segregated neighborhoods (Michigan Civil Rights Commission 2017).

Travel just 70 miles down I-75 from Flint to Detroit, Michigan, and we are able to witness in real time the way racial capitalism is shaping COVID-19 health inequities. In a report by Michigan’s Health Department, as of April 3, 2020, Detroit City and surrounding counties have the largest number of cases in the state; as Nichols (2020) wrote for the *New York Times*, Detroit is already mourning. Detroit and its surrounding areas have large populations of people of color, most of whom are Black Americans and populations that are poor and working class (Nichols, 2020; Schulz et al., 2002). Even more striking than the incidence rates, however, is statistics that reveal that out of the direct deaths related to COVID-19, 40% of them are of Black residents in a state that has only 14% Black population. The clock is already ticking in Detroit on the racial time bomb in the coronavirus crisis (Blow, 2020), and data replicate these trends in major metros across the United States including Chicago, New Orleans, and New York (McCarthy, 2020). The overrepresentation in mortality among Black Americans, or death gap, is a result of structural violence (Ansell, 2017) as created through a racial capitalist system. In the sections that follow, I detail how racial capitalism acts as a fundamental cause of health inequities and COVID-19.

## A. Racial Capitalism Influences Multiple Disease Outcomes

First, the people of Detroit already endure multiple health problems, such as high rates of diabetes (National Medical Association, 2015). An early report from Italy found that a large majority of COVID-19 fatalities occurred in those who had comorbidities, or additional illnesses like diabetes and asthma, that amplified COVID-19’s wear on the body (Ebhardt et al., 2020). To be clear, these racial differences in illnesses are not the result of biological or even behavioral differences in race but a result of racist, capitalist systems that structure people’s lives.

## B. Racial Capitalism Increases Multiple Disease Risk Factors

Racism and capitalism have, for example, mutually constructed racial residential segregation, which refers to the physical separation of groups into residential contexts that are patterned by race (Rothstein, 2017). Racial residential segregation has been

imposed by legislation, supported by major economic institutions, enshrined in the housing policies of the federal government, enforced by the judicial system, and legitimized by the ideology of white supremacy that was advocated by the church and other cultural institutions. (Williams & Collins, 2001, p. 405)

Residents in deprived neighborhoods have less access to green spaces and healthy, affordable foods; thus, restricting healthy behaviors. Racial residential segregation means poor people of color are also forced to live near manufacturing and other harmful toxins and wastes. People restricted to these areas endure multiple exposures to harmful physical and social environments and increased stressful events, all of which demonstrate how multiple risk factors shape health, including COVID-19.

A 2002 study by health researchers argued that racial and spatial relations were fundamental determinants of health in Detroit (Schulz et al., 2002). Not only is Detroit one of most segregated cities in America, as mapping data shows (Cable, 2013), but Detroit also ranks in the top 20 major cities in the United States with highest rates of homelessness (Frohlich, 2019), and the majority of those persons are Black. Homelessness is another way that racial capitalism puts the poor, older, and families of color at increased risk for consequences of COVID-19 (Torres, 2020). How can a person even shelter in place with no shelter?

Given our capitalist, privatized insurance system in the United States, most homeless and unemployed have inadequate access to quality health care (Ansell, 2017). Though, to be clear, America’s exceptionally unequal, extreme neoliberal health care system puts the entire country at risk (Gaffney, 2020). Health care inequities are another risk factor; the coronavirus does not have to discriminate across race and class, our health care system does that work on its own. Racial and economic differences in testing and treatment rates (Farmer, 2020) is one mechanism that shapes disparities. In a recent interview on PBS News Hour, Dr. Uché Blackstock shared the implications of racial bias in medical encounters:

when black and brown people interface with the health care system, they often encounter provider bias. So, we know, and it’s well-documented, that their pain is undertreated or their complaints are minimized. So, my concern is that, when these patients present to emergency departments and hospitals in their areas with COVID-19 symptoms, that their symptoms may be downplayed or they may not be taken seriously. And we do already have the data to support that trend continuing to happen. (Blackstock, 2020)

## C. Racial Capitalism Restricts Access to Flexible Resources That Buffer Negative Disease Outcomes

Additionally, racial capitalism is a fundamental cause because it shapes access to flexible resources. For example, those with high socioeconomic status secure a superior set of knowledge, power, money, power, prestige, and beneficial social connections, all of which can alleviate the consequences of the disease (Link & Phelan, 1995). Think about who has access to up-to-date reports of COVID-19 that communicate important health education facts on protection. The wealthy can also afford to pay others to do their grocery shopping or order online, meanwhile part-time Amazon workers forced to be on the front line write pleas about having no paid time off (Guendelsberger, 2020). And, why don’t employers value and protect the workers doing the essential jobs? Jason Hargrove, a Black bus driver in Detroit, who was exposed to COVID-19 and lacked access to proper safety equipment wondered this himself shortly before passing away from the disease (Witsil, 2020).

Racism also restricts those same crucial flexible resources, in addition to others even more racialized such as freedom. For instance, unfreedoms, or the lack of control Black Americans have over their lives in the United States, whether it be attributed to historical systems of slavery or mass incarceration today, puts them at heighted risks for mental and physical health problems (e.g., Alexander, 2020; Phelan & Link, 2018). The vulnerability and unfreedoms of detained populations at the border and in prisons, who are overwhelmingly Black and Brown and poor, increases their risk for harsh consequences of COVID-19 (Morse, 2020). Throughout Michigan’s prison system, as reported on April 3 by WXYZ Detroit, 184 incarcerated persons have already tested positive for COVID-19 (R. Jones, 2020).

## D. Racial Capitalism Shapes Disease Outcomes Overtime Despite Implementation of Intervening Mechanisms

Finally, intervening mechanisms found to mitigate some health inequities, like increased public sanitation or health education interventions, cannot fully eradicate the relationship between racism, poverty, and health because they are replaced by other mechanisms, like gentrification and rent surges that leads to housing instability and homelessness. In fact, mechanisms that sustain racial capitalism present a “fundamental resilience in the face of changing proximate causes” (Seamster & Ray, 2018, p. 330). The resilience can be evidenced in the revert back to previously mitigated mechanisms that are once again contributing to disease disparities, such as poor water sanitization (look again no further than the Flint water crisis).

Undeniably, racism and socioeconomic disadvantage have persistent, significant, multifaceted associations with poor health. Indeed, historical research on smallpox reveals that if access to flexible basic resources, like food, medicine, shelter, and treatment, excluded any subset of the population, the disease will continue to spread and continue to kill (Mitchell, 2020). During the 1918 flu epidemic in Chicago, racist tropes were used to blame the spread of the disease on Black residents, impeding a quality public health response (McDonald, 2020). History tells us that pandemics exacerbate race and class inequalities.

## COVID-19 Is Showing America Who We Are, Again—But What Can We Do?

An interview on Democracy Now with Dr. Abdul El-Sayed, former director of the Detroit Health Department, perfectly summarizes the impact of racial capitalism on COVID-19 inequities in the area, specifically highlighting political and economic decisions about water:

*when you look at communities that are suffering the most, they’re communities on which environmental injustice, structural racism, and their implications on poverty, have already softened the space for the incoming of this virus to devastate people* [emphasis added]. You know, you think about something like water . . . Detroiters were literally having to pay back the debt that the entire region incurred because Detroit was the single utility purifying water for everybody. And then they just raised rates . . . and then you fast-forward, and you think about the incoming pandemic, and we’re telling people to wash their hands with warm, soapy water for 20 seconds. Well, if you don’t have water in your house, you can’t do that. All of those—*all of that is seeded by decisions that have been made, that have been patterned around race and patterned around wealth for a very long time* [emphasis added]. (El-Sayed, 2020)

Racialized capitalist pursuits have left behind the poor, people of color in Detroit, devaluing life so much that it is being easily snatched up by the novel coronavirus pandemic.

Speaking even more broadly, capitalist gain has threatened the health of millions of Americans within the pandemic. Volunteer innovators printing important medical technologies report being threatened with litigation from large corporations (Peters, 2020). Individual racketeers have wiped out entire city’s stock of hand sanitizer in seek of profit. Wisconsin Republicans in power reject extensions for returning absentee ballots exploiting the pandemic and increasing voter disenfranchisement of poor, people of color (Bearman, 2020). Xenophobic racism has already affected health outcomes, as governmental officials lacked to enact policies by othering the problem, or later enacting targeted border policies (Goh, 2020). Anti-Asian interpersonal discrimination has increased and social distancing may spike rates of White Nationalism (Dickson, 2020). “Each public health issue is a snapshot where we can see the unfolding of the collective processes that define who we are, what we believe, and what we value as a society” (Wallack, 2019, p. 901). *COVID-19 is showing us who we are . . . again.*

The racist, capitalist frameworks that sustain the modern world is a fundamental cause of COVID-19 within and across countries, but what can we do? As a collective, we must first ask, what would it take to create the change we need to solve this problem (Wallack, 2019)? Public health and health education research, in particular, must look beyond interventions focused to individual and interpersonal characteristics and more to institutions, environments, and ideologies (Golden & Earp, 2012). As Link and Phelan (1995) instruct, “If one wishes to address fundamental social causes, the intervention must address inequality in the resources that fundamental causes entail” (p. 89). C. P. Jones (2014) offers three tangible way to address health equity that combats racial capitalism: “valuing all individuals and populations equally; recognizing and rectifying historical injustices; providing resources according to need.” (p. S75).
